# Supplementary material for: Mucin-binding protein shuttles enable delivery of brain-targeted therapeutics
Source: bioRxiv. 2026 Apr 12:2026.03.22.713512. Originally published 2026 Mar 25. Preprint. [Version 2] doi: 10.64898/2026.03.22.713512 (PMC13041825; doi:10.64898/2026.03.22.713512)
Supplement: 1 [file NIHPP2026.03.22.713512v2-supplement-1.pdf]

bioRxiv preprint doi: https://doi.org/10.64898/2026.03.22.713512; this version posted April 12, 2026. The copyright holder for this preprint (which was not certified by peer review) is the author/funder, who has granted bioRxiv a license to display the preprint in perpetuity. It is made available under aCC-BY 4.0 International license.

Supplementary Figures

bioRxiv preprint doi: https://doi.org/10.64898/2026.03.22.713512; this version posted April 12, 2026. The copyright holder for this preprint (which was not certified by peer review) is the author/funder, who has granted bioRxiv a license to display the preprint in perpetuity. It is made available under aCC-BY 4.0 International license.

a

GlycoShuttle	Structure	pI	MW (kDa)	Glycan Specificity
SMS1 (StcE^{E447D})	monomer	6	98	N mucin domain C
SMS2	monomer	9	11	N mucin domain C
WGA	dimer	9	38	Terminal GlcNAc sialic acid
ConA	tetramer	5	104	branched and terminal mannose
SNA	tetramer	5.5	140	2,6-linked sialic acid
VVA	tetramer	6	144	Terminal GalNAc (Tn antigen)
PNA	tetramer	6	110	Terminal Gal (T antigen)

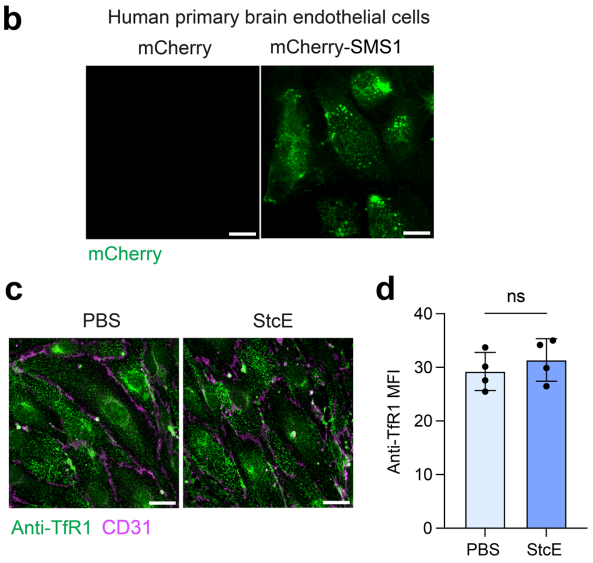

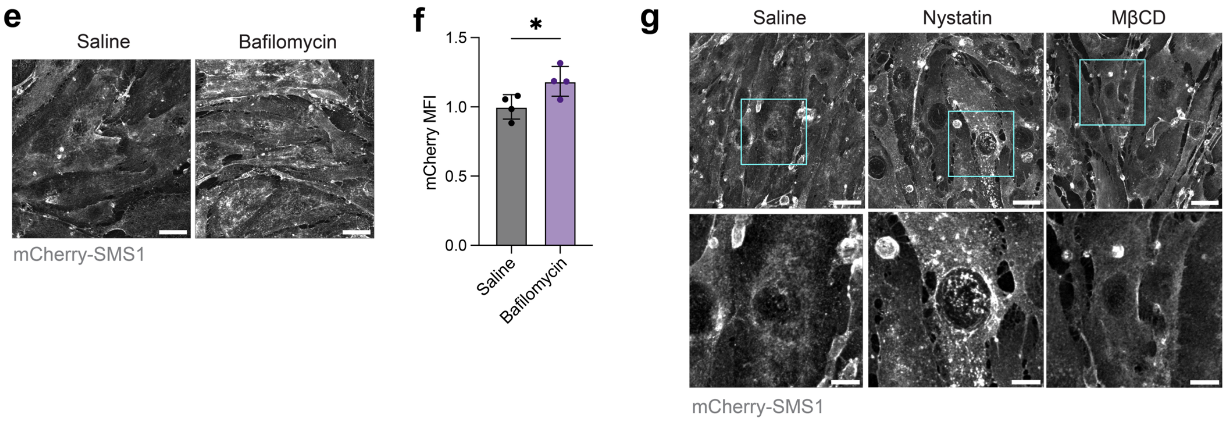

Fig. S1. Characterization of GlycoShuttle constructs.

a) Properties of glycocalyx-binding proteins tested including quaternary structure, molecular weight (MW), isoelectric point (pI), and glycan specificity *(9)*.
b) SMS1 shuttles mCherry cargo into human primary brain endothelial cells. Scale bar=25 µm.
c) Anti-TfR1 (8D3) internalization into bEnd.3 cells is unaffected by StcE-mediated mucin removal. Scale bar=25 µm.
d) Quantification of anti-TfR1 MFI in (c) (n=4 wells per construct; two-sided t-test; mean ± s.e.m.).
e) Treatment of bEnd.3 cells with bafilomycin increases mCherry-SMS1 punctate signal. Scale bar=25 µm.
f) Quantification of mCherry MFI in (f) (n=4 wells per construct; two-sided t-test; mean ± s.e.m.).
g) Treatment of bEnd.3 cells with caveolae/lipid raft inhibitors perturbs mCherry-SMS1 binding and internalization into cells. Scale bar=25 µm.

bioRxiv preprint doi: https://doi.org/10.64898/2026.03.22.713512; this version posted April 12, 2026. The copyright holder for this preprint (which was not certified by peer review) is the author/funder, who has granted bioRxiv a license to display the preprint in perpetuity. It is made available under aCC-BY 4.0 International license.

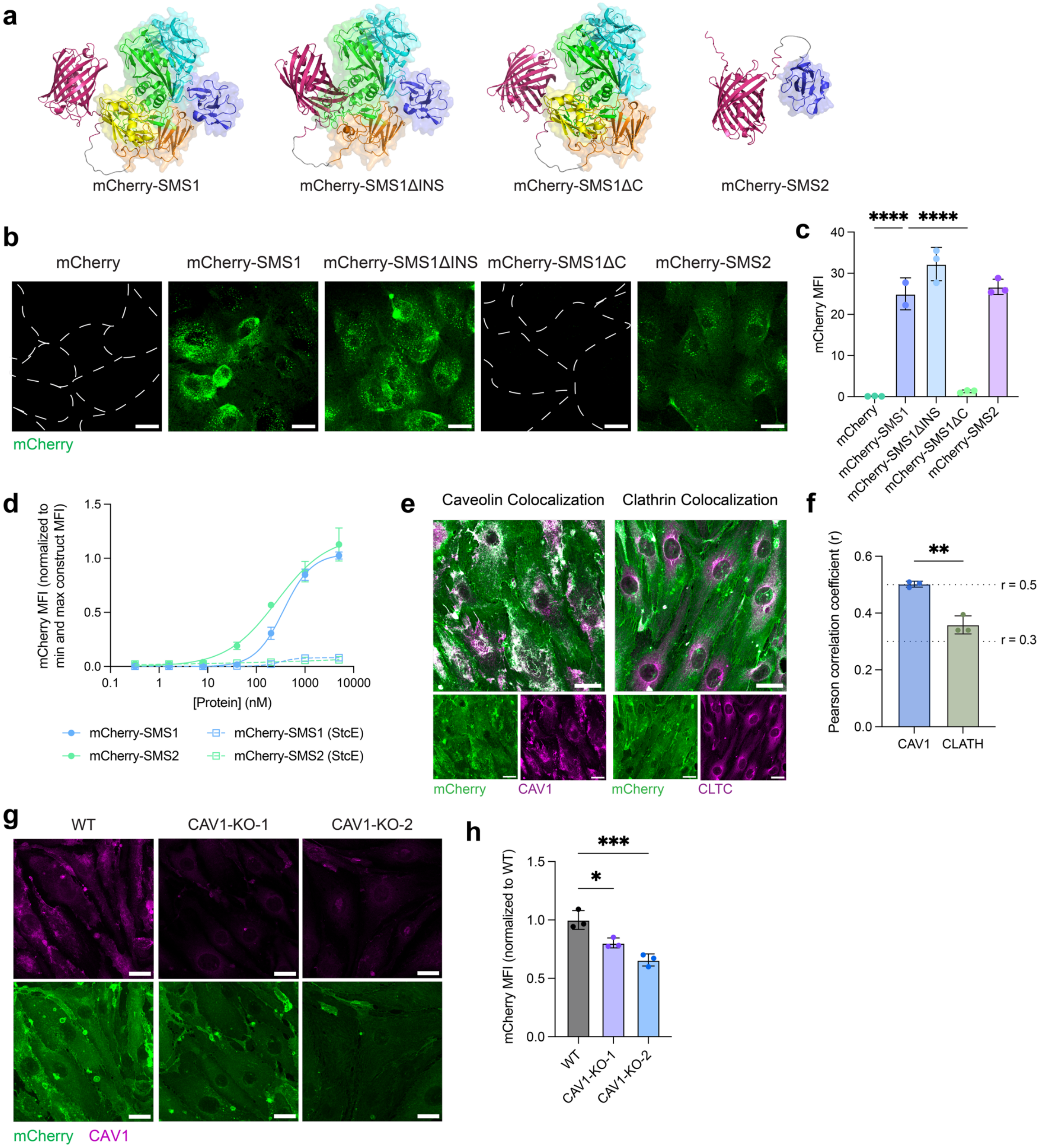

Fig. S2. Characterization of mCherry-SMS variants.

a) Structures of mCherry-SMS variants, as predicted by AlphaFold.
b) Binding and internalization of mCherry-SMS1, mCherry-SMS1ΔINS, mCherry-SMS1ΔC, mCherry-SMS2, and mCherry in human primary brain endothelial cells after 30-min incubation at 37°C. Cell boundaries for mCherry-SMS1ΔC and mCherry are marked in white dotted line. Scale bar=25 µm.
c) Quantification of (b) (n=2-3 wells per construct; one-way ANOVA with Dunnett's post hoc test; mean ± s.e.m.).

bioRxiv preprint doi: https://doi.org/10.64898/2026.03.22.713512; this version posted April 12, 2026. The copyright holder for this preprint (which was not certified by peer review) is the author/funder, who has granted bioRxiv a license to display the preprint in perpetuity. It is made available under aCC-BY 4.0 International license.

d) Binding curves of mCherry-SMS1 and mCherry-SMS2 on HeLa cells with and without StcE treatment.
e) mCherry-SMS2 colocalization with CAV1 and CLTC in bEnd.3 cells with colocalization threshold mask in grayscale. Scale bar=25 µm.
f) Colocalization analysis using Pearson correlation between mCherry-SMS2 and CAV1 or CLTC in bEnd.3 cells (n=3 wells per construct; two-sided t-test; mean ± s.e.m.).
g) mCherry-SMS2 internalization in wild-type (WT) and two CAV1-KO cell lines. Scale bar=25 µm.
h) Quantification of mCherry MFI in (g) (n=3 wells per construct; one-way ANOVA with Dunnett's post hoc test; mean ± s.e.m.).

bioRxiv preprint doi: https://doi.org/10.64898/2026.03.22.713512; this version posted April 12, 2026. The copyright holder for this preprint (which was not certified by peer review) is the author/funder, who has granted bioRxiv a license to display the preprint in perpetuity. It is made available under aCC-BY 4.0 International license.

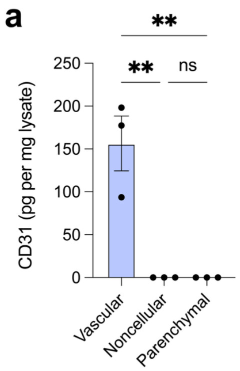

Fig. S3. Validation of vascular depletion.

a) CD31 measurement in vascular cell, non-cellular, and parenchymal cell fractions by ELISA after vascular depletion (n=3 animals; one-way ANOVA with Tukey's post hoc test; mean ± s.e.m.).

bioRxiv preprint doi: https://doi.org/10.64898/2026.03.22.713512; this version posted April 12, 2026. The copyright holder for this preprint (which was not certified by peer review) is the author/funder, who has granted bioRxiv a license to display the preprint in perpetuity. It is made available under aCC-BY 4.0 International license.

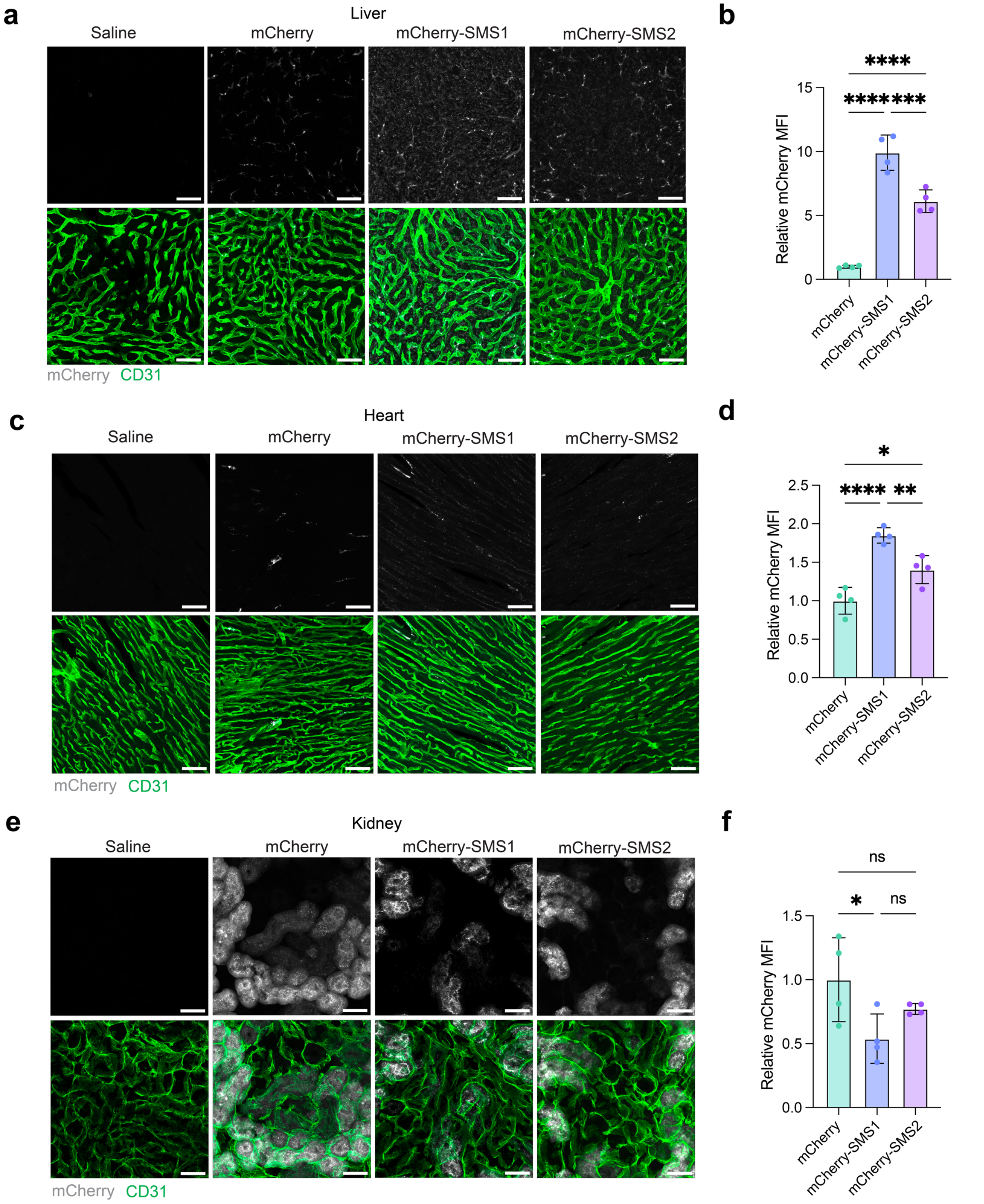

Fig. S4. Distribution of mCherry-SMS1 and mCherry-SMS2 in peripheral organs.

a) Representative liver sections showing distribution of mCherry, mCherry-SMS1, and mCherry-SMS2 at 48 hours following equimolar dosing (equivalent to 5 mg/kg mCherry-SMS1). Scale bar=50 μm.

bioRxiv preprint doi: https://doi.org/10.64898/2026.03.22.713512; this version posted April 12, 2026. The copyright holder for this preprint (which was not certified by peer review) is the author/funder, who has granted bioRxiv a license to display the preprint in perpetuity. It is made available under aCC-BY 4.0 International license.

b) Quantification of relative mCherry MFI in (a) (n=4 animals per construct; one-way ANOVA with Tukey's post hoc test; mean ± s.e.m.).
c) Representative heart sections showing distribution of mCherry, mCherry-SMS1, and mCherry-SMS2 at 48 hours following equimolar dosing (equivalent to 5 mg/kg mCherry-SMS1). Scale bar=50 µm.
d) Quantification of relative mCherry MFI in (c) (n=4 animals per construct; one-way ANOVA with Tukey's post hoc test; mean ± s.e.m.).
e) Representative kidney sections showing distribution of mCherry, mCherry-SMS1, and mCherry-SMS2 at 48 hours following equimolar dosing (equivalent to 5 mg/kg mCherry-SMS1). Scale bar=50 µm.
f) Quantification of relative mCherry MFI in (e) (n=4 animals per construct; one-way ANOVA with Tukey's post hoc test; mean ± s.e.m.).

bioRxiv preprint doi: https://doi.org/10.64898/2026.03.22.713512; this version posted April 12, 2026. The copyright holder for this preprint (which was not certified by peer review) is the author/funder, who has granted bioRxiv a license to display the preprint in perpetuity. It is made available under aCC-BY 4.0 International license.

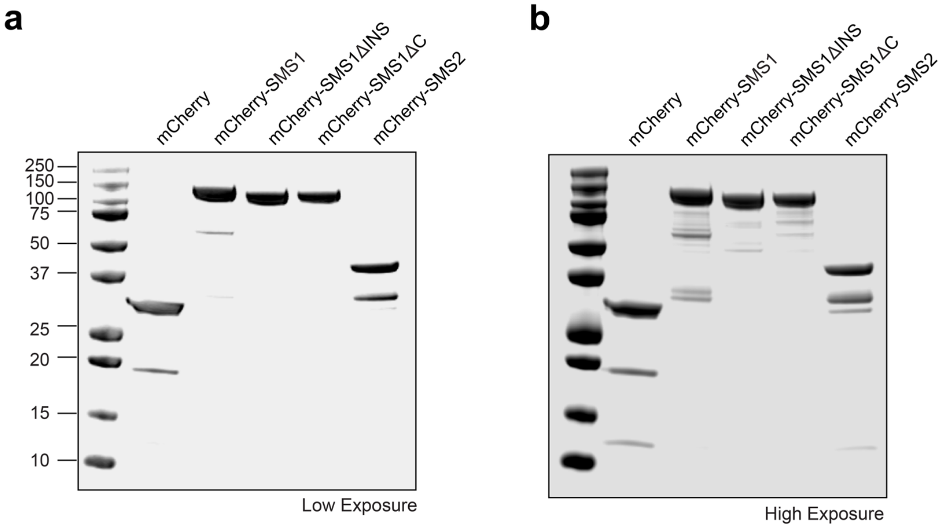

Fig. S5. Validation of mCherry-SMS variants.

a) SDS-PAGE gel of mCherry-SMS variants with low exposure.
b) SDS-PAGE gel of mCherry-SMS variants with high exposure.

bioRxiv preprint doi: https://doi.org/10.64898/2026.03.22.713512; this version posted April 12, 2026. The copyright holder for this preprint (which was not certified by peer review) is the author/funder, who has granted bioRxiv a license to display the preprint in perpetuity. It is made available under aCC-BY 4.0 International license.

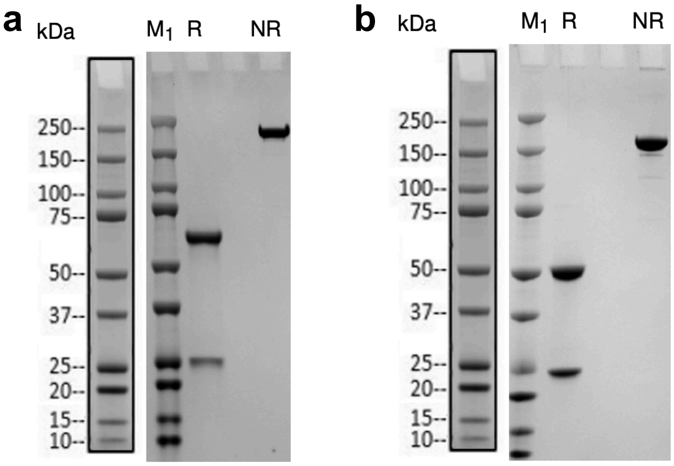

Fig. S6. Validation of anti-BACE1 antibody constructs.

a) SDS-PAGE gel of anti-BACE1-SMS2 from Genscript. R: Reducing condition; NR: Non-reducing condition; M_1: Protein ladder.
b) SDS-PAGE gel of anti-BACE1 from Genscript. R: Reducing condition; NR: Non-reducing condition; M_1: Protein ladder.

bioRxiv preprint doi: https://doi.org/10.64898/2026.03.22.713512; this version posted April 12, 2026. The copyright holder for this preprint (which was not certified by peer review) is the author/funder, who has granted bioRxiv a license to display the preprint in perpetuity. It is made available under aCC-BY 4.0 International license.

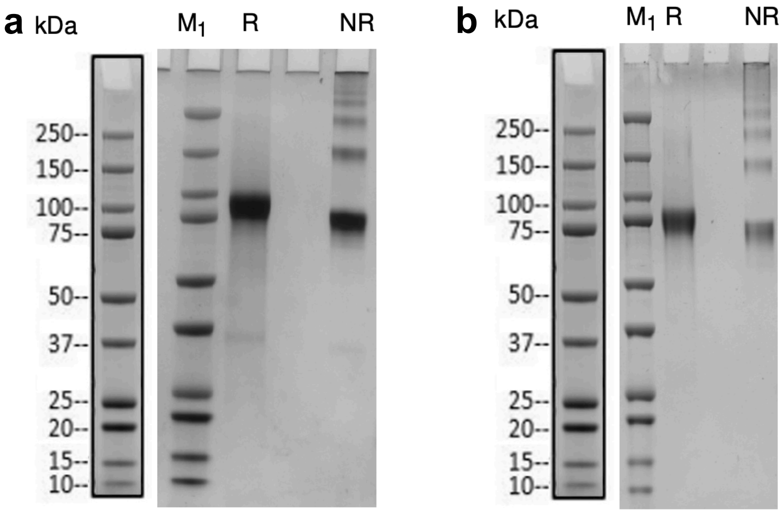

Fig. S7. Validation of progranulin constructs.

a) SDS-PAGE gel of SMS2-hPGRN from Genscript. R: Reducing condition; NR: Non-reducing condition; M_1: Protein ladder.
b) SDS-PAGE gel of hPGRN from Genscript. R: Reducing condition; NR: Non-reducing condition; M_1: Protein ladder.
